# Mechanical ventilation parameters in critically ill COVID-19 patients: a scoping review

**DOI:** 10.1186/s13054-021-03536-2

**Published:** 2021-03-20

**Authors:** Giacomo Grasselli, Emanuele Cattaneo, Gaetano Florio, Mariachiara Ippolito, Alberto Zanella, Andrea Cortegiani, Jianbo Huang, Antonio Pesenti, Sharon Einav

**Affiliations:** 1 Department of Pathophysiology and Transplantation, University of Milan, Milan, Italy; 2 Dipartimento Di Anestesia, Rianimazione ed Emergenza-Urgenza, Fondazione IRCCS Ca’ Granda Ospedale Maggiore Policlinico, Via Francesco Sforza 35, 20122 Milan, Italy; 3 Department of Surgical, Oncological and Oral Science (Di.Chir.On.S.), University of Palermo, Palermo, Italy; 4 Department of Anesthesia, Intensive Care and Emergency, Policlinico Paolo Giaccone, University of Palermo, Palermo, Italy; 5 Department of General Surgery, The First Affiliated Hospital, Chongqing Medical University, Chongqing, China; 6 General Intensive Care Unit, Shaare Zedek Medical Center, Jerusalem, Israel; 7 Faculty of Medicine, Hebrew University, Jerusalem, Israel

**Keywords:** Coronavirus disease 2019, Acute respiratory distress syndrome, Mechanical ventilation, Intensive care units

## Abstract

**Background:**

The mortality of critically ill patients with COVID-19 is high, particularly among those receiving mechanical ventilation (MV). Despite the high number of patients treated worldwide, data on respiratory mechanics are currently scarce and the optimal setting of MV remains to be defined. This scoping review aims to provide an overview of available data about respiratory mechanics, gas exchange and MV settings in patients admitted to intensive care units (ICUs) for COVID-19-associated acute respiratory failure, and to identify knowledge gaps.

**Main text:**

PubMed, EMBASE, and MEDLINE databases were searched from inception to October 30, 2020 for studies providing at least one ventilatory parameter collected within 24 h from the ICU admission. The quality of the studies was independently assessed using the Newcastle–Ottawa Quality Assessment Form for Cohort Studies. A total of 26 studies were included for a total of 14,075 patients. At ICU admission, positive end expiratory pressure (PEEP) values ranged from 9 to 16.5 cm of water (cmH_2_O), suggesting that high levels of PEEP were commonly used for setting MV for these patients. Patients with COVID-19 are severely hypoxemic at ICU admission and show a median ratio of partial pressure of arterial oxygen to fraction of inspired oxygen (PaO_2_/FiO_2_) ranging from 102 to 198 mmHg. Static respiratory system compliance (Crs) values at ICU admission were highly heterogenous, ranging between 24 and 49 ml/cmH_2_O. Prone positioning and neuromuscular blocking agents were widely used, ranging from 17 to 81 and 22 to 88%, respectively; both rates were higher than previously reported in patients with “classical” acute respiratory distress syndrome (ARDS).

**Conclusions:**

Available data show that, in mechanically ventilated patients with COVID-19, respiratory mechanics and MV settings within 24 h from ICU admission are heterogeneous but similar to those reported for “classical” ARDS. However, to date, complete data regarding mechanical properties of respiratory system, optimal setting of MV and the role of rescue treatments for refractory hypoxemia are still lacking in the medical literature.

**Supplementary Information:**

The online version contains supplementary material available at 10.1186/s13054-021-03536-2.

## Introduction

Mechanical organ support has always been a mainstay of intensive care and especially the use of mechanical ventilation. Among the more than 70 million people infected worldwide with SARS-CoV-2, many have required mechanical ventilation [1, 2]. Questions are being asked regarding the “correct” mode of ventilation for these patients and to date no literature review has been published on the topic.

Approximately one in ten patients with SARS-CoV-2 becomes symptomatic [3]. Although hospital and Intensive Care Unit (ICU) admission rates are highly dependent on resource availability, most studies from Europe and North America report that 10–20% (depending on age) of the patients admitted to hospital undergo some form of mechanical ventilatory support due to acute hypoxemic respiratory failure, either in the ward or in the ICU [4, 5]. Overall, between one-fourth and one-third of hospitalized patients will ultimately be admitted to the ICU [6, 7].

The mortality of patients with critical coronavirus disease 2019 (COVID-19) is strikingly high, ranging between 15 [8] and 74% [9], particularly when invasive mechanical ventilation (IMV) has been required. Consequently, questions have been raised regarding the relationship between various aspects of mechanical ventilation and patient outcomes in this scenario. One of the pinnacles of intensive care achievements in the last two decades has been the recognition that inappropriate setting of mechanical ventilation is a major contributor to lung damage (so called ventilator-induced lung injury (VILI)) in patients with “classical” acute respiratory distress syndrome (ARDS) [10–14]. However, there is ongoing discussion regarding the relevance of this insight to the outcomes of patients with SARS-CoV-2. The first step required to resolve this question is to summarize the currently available data. This scoping review aimed to map the existing information regarding the respiratory mechanics, mechanical ventilation settings and parameters of gas exchange in critically ill patients undergoing invasive mechanical ventilation (IMV) for treatment of severe COVID 19 and to identify knowledge gaps.

## Methods

The review was prospectively registered in the Open Science Framework (OSF) (August 18, 2020; osf.io/8grfc) and was conducted in accordance with the Preferred Reporting Items for Systematic reviews and Meta-Analyses extension for Scoping Reviews (PRISMA-ScR) [15]. The filled PRISMA-ScR checklist is provided in Additional file 1. We aimed to study respiratory mechanics, ventilation settings and parameters of gas exchange reported in adult critically ill patients with COVID-19 undergoing invasive mechanical ventilation in ICUs. We excluded studies reporting data on children and on adult patients undergoing non-invasive ventilation. The inclusion and exclusion criteria are summarized in Table S3 (Additional file 2).

### Search strategy

Two authors (AC, EC) developed the search strategy, which is reported in full in Additional file 2. PubMed, EMBASE, and MEDLINE databases were searched from inception to October 30, 2020 for English-only articles. Following the initial search, three of the reviewers (EC, GF, AZ) independently screened the titles and abstracts of the retrieved papers to identify those warranting full review. Studies reporting duplicate patient populations were excluded. Two of the authors (EC, GF) accessed the selected papers for full-text review and evaluated them for inclusion. We included only original articles (non-randomized studies, excluding case reports) reporting at least one parameter pertinent to our study question (i.e. respiratory mechanics, ventilation settings, gas exchange at ICU admission or within the first day of ICU stay). This time frame was selected based on the understanding that most critically ill patients undergo routine baseline assessments at admission. Articles not reporting any baseline ventilation parameter and those including only patients treated with extracorporeal membrane oxygenation (ECMO) were excluded at this stage. The final selection included only studies reporting at least one ventilatory parameter as detailed below (see “data extraction”).

### Quality of the studies

Two authors (AC, EC) independently assessed the quality of the studies using the Newcastle–Ottawa Quality Assessment Form for Cohort Studies [16]. A third author (GG) resolved discrepancies at any stage.

### Data extraction

We extracted the data presented on each of the following respiratory parameters from the included papers: positive end-expiratory pressure (PEEP), Tidal Volume in relation to predicted body weight (TV/Pbw), plateau pressure (Pplat), driving pressure (ΔP), static compliance of the respiratory system (Crs), respiratory rate (RR) and mechanical power. As noted above, only the baseline measurements, (i.e. those recorded within the first 24 h of ICU stay) were charted. We also collected data on gas exchange parameters including partial pressure of arterial oxygen (PaO_2_), ratio of partial pressure of arterial oxygen to fraction of inspired oxygen (PaO_2_/FiO_2_), partial pressure of arterial carbon dioxide (PaCO_2_), and use of rescue therapies (neuromuscular blocking agents, prone positioning, inhaled pulmonary vasodilators, extracorporeal membrane oxygenation) for refractory hypoxemia when available. All data were extracted by one author (EC), using a standardized Excel form (Microsoft Excel™ Version 2016 for Windows). A second author (GF) verified and validated the charted data. In most studies the data were reported as medians with their interquartile ranges (IQRs), while in others they were reported as means with their standard deviations (SDs). For the purpose of this review we report categorical variables as counts and percentages, and continuous data as means (± SDs) or medians (± IQRs) as presented in the original reports.

## Results

The search strategy initially identified 6460 potentially relevant papers. After removal of duplicates, the titles and abstracts of 6458 papers were screened of which 6401 were excluded and 57 were selected for full text review. Among the 57 papers undergoing full review, 31 papers were subsequently excluded—21 for not reporting any of the required parameters, 8 for reporting relevant data outside of the predetermined time frame and 2 for describing only patients treated with ECMO.

**Fig. 1:**
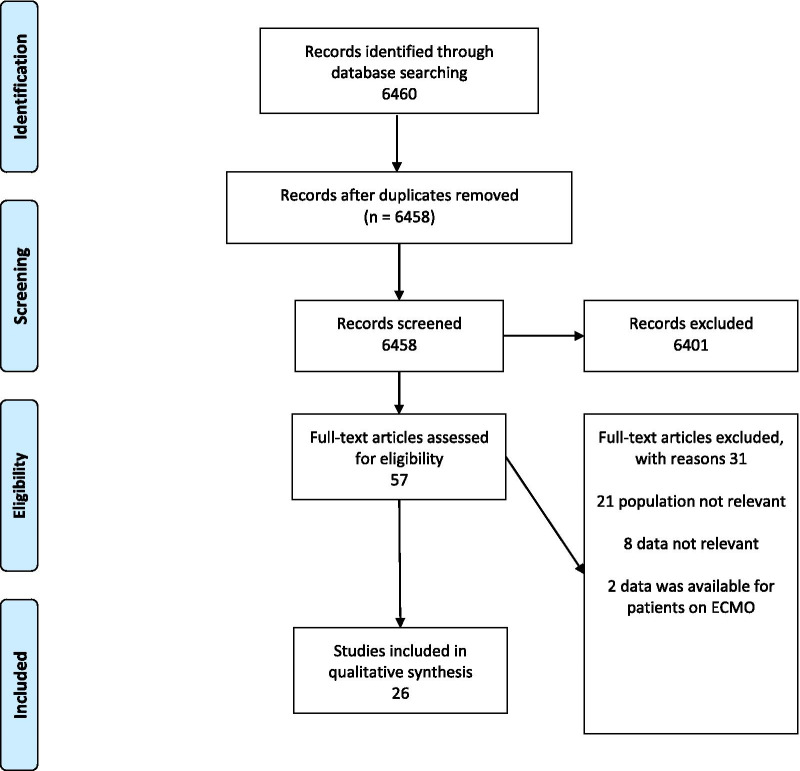
PRISMA flow diagram of included studies

The final selection included 26 studies: 4 multicenter prospective cohort studies [17–20], 6 multicenter retrospective cohort studies [8, 21–25], 1 single center prospective cohort study [26], 1 multicenter prospective case series [27], 2 multicenter retrospective case series [28, 29], 4 single center prospective case series [30–33] and 8 single center retrospective case series [34–41] (Fig. 1).

**Fig. 2:**
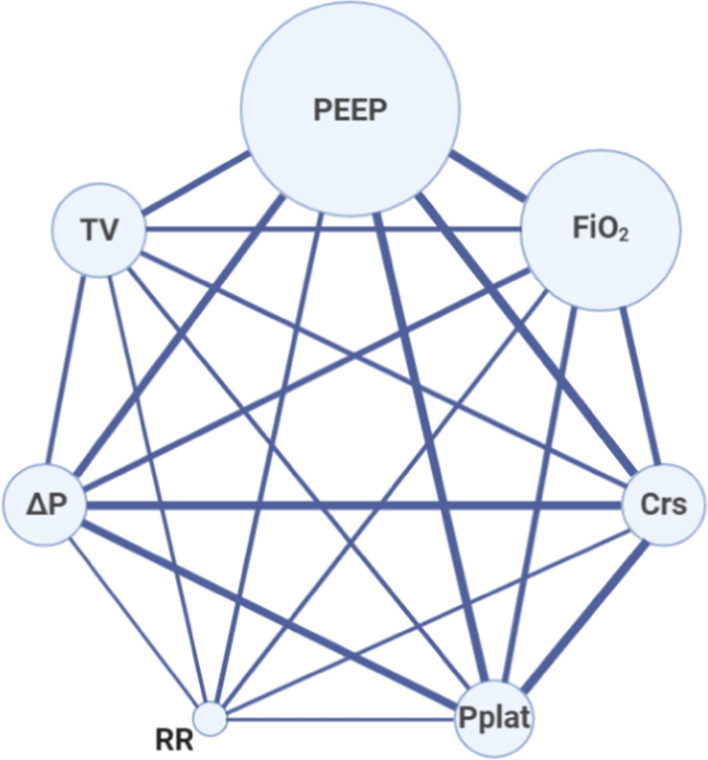
Network geometry shows nodes as reported respiratory mechanic parameters or ventilator settings and studies reporting a couple of parameters as lines. The size of the nodes is proportional to the number of patients with that reported parameter. The thickness of the connecting line is proportional to the number of studies that report both the connected parameter. Abbreviations: PEEP, positive end-expiratory pressure; FiO_2_, fraction of inspired oxygen; Crs, compliance of the respiratory system; Pplat, plateau pressure; RR, respiratory rate; ΔP, driving pressure; TV, tidal volume

Table S1 (see Additional file 2) summarizes the main characteristics of the included studies. Most of the studies described patients receiving mechanical ventilation in either North America or Europe (25/26), and one study was conducted in China. As shown in Table 1, the number of respiratory mechanics and ventilation settings parameters provided by the studies was very heterogeneous. Two of the largest studies [21, 22] reported only PEEP values, while other studies provided a more complete set of data at ICU admission (Fig. 2).

**Table 1:** Number of patients and ventilation characteristics described in the included studies

Study	Sample size, no.	Tidal volume ml/kg	Respiratory rate breath/min	PEEP cmH_2_O	Plateau pressure cmH_2_O	Driving pressure cmH_2_O	Crs mL/cmH_2_O	Mechanical power J/min	FiO_2_%	PaO_2_/FiO_2_ ratio mmHg	PaCO_2_ mmHg
Schmidt [17]	4643	6.1 [5.8–6.7]		12 [10–14]	24 [21–27]	13 [10–17]	33 [26–42]	26.5 [19–35]		154 [106–223]	40 [35–46]
Grasselli [22]	3988			13 [10–15]					70 [60–85]	145 [103–203]	
Gupta [21]	2215			12 [10–15]					80 [60–100]	124 [86–188]	
Ferrando [19]	742	6.9 [6.3–7.8]	24 [20–30]	12 [11–14]	25 [22–29]	12 [10–16]	35 [27–45]		80 [60–100]	120 [83–177]	45 [37–55]
Botta [23]	553	6.3 [5.7–7.1]	20 [18–24]	14 [11–15]		14 [11–16]	31.9 [26–39.9]	17.7 [14–22]	60 [50–80]	159 [129–201]	44 [38–51]
Grasselli [18]	301	7 [6.3–7.6]	20 [18–24]	13 [10–15]	24 [22–26]	11 [9–14]	41 [33–52]	16.8 [14–21]	60 [50–80]	124 [89–164]	46 [40–53]
Schenck [26]	267	7.01 [6.13–8.1]		10 [8–12]	25 [21–29]	14 [11–17]	28 [23–38]			103 [82–134]	44 [38–52]
Cummings [20]	257	6.2 [5.9–7.2]		15 [12–18]	27 [23–31]	15 [11–18]	27 [22–36]		100 [80–100]	129 [80–203]	
Roedl [24]	223		22 [20–28]	12 [10–15]					60 [50–80]		
Auld [25]	217						34 [28–46]			132 [100–178]	
Mitra [8]	117			12 [10–14]	22 [20–24]		35 [31–44]		50 [40–60]	180 [148–216]	
Pandya [35]	75			10.3 (3.9)	24.4 (6.7)	14.3 (6.1)	37.8 (21.9)		62 (32)	162 [106–316]	
Cavayas [41]	75	7.5 [6.8–8.7]	20 [16–22]	9 [8–10]	21 [19–24]	13 [10–16]	48 [38–58]	20.3 [16–28]	50 [40–65]	177 [138–276]	44 [40–49]
Zangrillo [36]	73	6.7 [6–7.5]		12 [10–14]		12 [7–16.5]			70 [52–80]	110 [80–158.5]	46.4 [40.0–51.3]
Ziehr [28]	66			10 [8–12]	21 [19–26]	11 [9–12]	35 [30–43]			182 [135–245]	
Sinha [27]	39			12 [6–20]	31 [27–34]		24 [20–28]			135 [113–158]	
Laverdure [37]	36	6.1 (0.6)	25 [24–27]	13.4 (3.2)			39.4 (16.9)		65 [50–100]	152 [112–240]	
Bos [34]	38			10 [9–12]	20.5 [17–23]	10.5 [7–13]	49 (24.5)			131.8 [47.9]	
Haudebourg [30]	30	6 [5.9–6.7]	28 [28–30]	10 [8–12]	21 [20–24]	10 [8–12]	44 [35–51]		70 [52–80]	119 [97–163]	
Beloncle [31]	25	6.0 [5.9–6.1]	28 [26–30]	12 [10–15]	23 [21–24]				60 [40–65]	135 [119–195]	41 [38–44]
Bhatraju [29]	24				25 [20–28]	13 [11–17]	29 [25–36]		90 [70–100]	142 [94–177]	
Diehl [32]	22		33 [28.5–35]	16.5 [16–18]	27 [25–28]	9.5 [9–11.75]	39.5 [33–45]		45 [40–58]	198 [167–298]	55 [44–62]
Pedersen [38]	17	5.6 (1)							62 (20)		44.2 (8.2)
Roesthuis [33]	14				24 (3)	11 (2)	42 (3)		57 (15)	162 (48)	57 (13)
Carsetti [39]	10			14 (1,49)	24 (2)	9.5 (3)	49 (9)		70 (0.18)	119 (33.6)	
Liu X [40]	8	7.5 (0.6)	20.1 (1.5)	9.6 (1.2)	23.6 (3)	14 (2.5)	33.9 (7.6)			102 (27.9)	41.8 (3.7)
LUNG SAFE [50]	2377	7.6 [7.5–7.7]	20.8 [21.2–21.5]	8.4 [8.3–8.6]	23.2 [23, 24]		32 [25–43]		0.65 [0.64–0.65]	161 [158–163]	46 [45.4–46.6]

Data are number, median [IQR], or mean (SD). PEEP, positive end expiratory pressure; Crs, compliance of the respiratory system; FiO_2_, fraction of inspired oxygen; PaO_2_ partial pressure of arterial oxygen; PaCO2 partial pressure of arterial carbon dioxide

Table S2 (see Additional file 2) reports the quality of the included studies as assessed using the Newcastle–Ottawa Quality Assessment tool and shows that all studies except one are of poor quality.

### Respiratory mechanics and ventilation settings within the first 24 hours of ICU stay

*Mode of ventilation:* The mode of ventilation was reported in 5/26 studies. In four of them the choice was volume-controlled ventilation [18, 26, 31, 36] while in the fifth study pressure-controlled ventilation was used in 52% and volume-controlled ventilation in 19% of the cases [23].

*Tidal volume:* TV was reported in 13/26 studies. The values of TV per predicted body weight varied from 5.6 to 7.5 ml/Kg [38, 40, 41].

*Respiratory rate:* RR was reported in 10/26 studies and ranged from 20 to 33 breaths/min [18, 23, 32, 41].

*PEEP:* All but three of the studies reported PEEP with median values that ranged from a minimum of 9 cmH_2_O [41] to a maximum of 16.5 cmH_2_O [32]; only two of the studies reported a median value lower than 10 cmH_2_O.

*Plateau pressure:* Plateau pressures, which were reported in 18/26 studies ranged from 20.5 to 31 cmH_2_O [27, 34].

*Driving pressure:* Driving pressures were reported in 17/26 studies and ranged from 9.5 to 15 cmH_2_O [20, 32, 39].

**Fig. 3:**
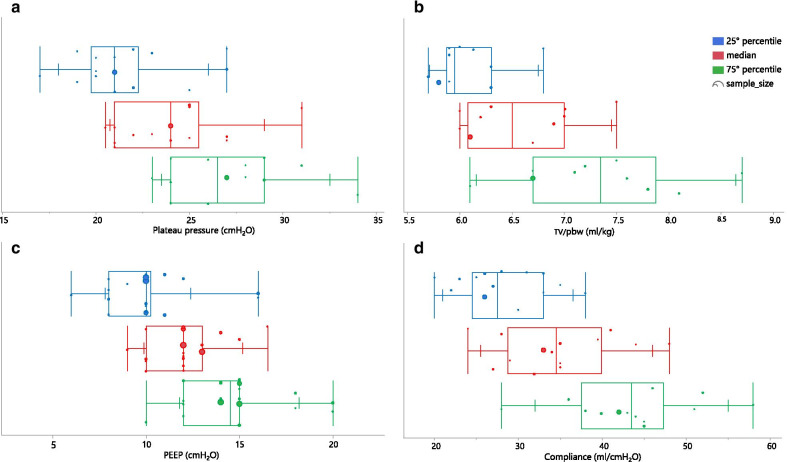
Multi-panel figure showing the distribution of the reported median, 25th and 75th percentile of **a**: plateau pressure; **b**: Tidal Volume/Pbw; **c**: PEEP; **d**: respiratory system compliance. Each circle represents a single study and its size is proportional to the number of included patients. (abbreviation: PEEP positive end expiratory pressure; TV/pbw Tidal Volume/predicted body weight)

*Static compliance:* Static respiratory system compliance was reported in 20/26 studies. The values reported showed wide variability, ranging from 24 [27] to 49 ml/cmH_2_O [34, 39] (Fig. 3), although the range was slightly more narrow, from 27 to 41 ml/cmH_2_O, in studies that included more than 100 patients [18, 20].

*Mechanical Power:* Mechanical Power was reported in only 4/26 studies. The median values reported ranged from 26.5 [17] to 16.8 J/min [18].

### Gas exchange within the first 24 hours of ICU stay

PaO_2_ values were reported in only 7/26 studies. The reported values ranged from 73 to 95 mmHg [22, 38].

PaCO_2_ values were reported in 12/26 studies and varied between 40 and 57 mmHg [17, 33].

FiO_2_ was reported in 19/26 studies, and ranged from 45 to 100% [20, 32].

PaO_2_/FiO_2_ was reported in 24/26 studies. All patients had acute hypoxemic respiratory failure, with a median PaO_2_/FiO_2_ that ranged from 102 to 198 mmHg [32, 40].

When considering only studies with more than 100 patients, median values of the PaO_2_/FiO_2_ ratio and PaCO_2_ ranged from 103 to 180 mmHg [8, 26] and 40 to 46 mmHg [17, 18], respectively.

### Patient management

Table 2 summarizes other aspects of respiratory patient management during ICU stay, including the use of rescue therapies for ARDS (data from 16/26 papers), and clinical outcomes (reported in 20/26 papers).

**Table 2:** Patient management strategies and outcomes in the included studies

Study	Invasive Mechanical Ventilation	NMBA	Prone Positioning	Pulmonary vasodilator	ECMO	Death	Still in ICU	Discharged from ICU
Schmidt [17]	3376/4643 (72%)	1966/2224 (88%)	1556/2223 (70%)	425/2224 (19%)	235/2153 (11%)	1298/4244 (31%)		
Gupta [21]	1859/2215 (84%)	909/1859 (49%)	852/1859 (46%)	212/1859 (11%)	61/1859 (3.3%)	875/2215 (39.5%)		
Grasselli [22]	2929/3355 (87%)				64/3857 (1.7%)	1769/3988 (44%)	91/3988 (2.3%)	2049/3988 (51%)
Ferrando [19]	742/742 (100%)	536/742 (72%)	564/735 (77%)		21/738 (2.8%)	241/742 (32%)	100 (13%)	401/742 (54%)
Botta [23]	553/553 (100%)	183/487 (38%)	283/530 (53%)		2/553 (< 1%)	203/530 (38%)		
Grasselli [18]	301/301 (100%)					93/261 (35.6%)		
Schenck [26]	267/267 (100%)	161/267 (60%)	108/267 (40%)			49/267 (18%)	140/267 (52%)	
Cummings [20]	203/257 (79%)	51/203 (25%)	35/203 (17%)	22/203 (11%)	6/203 (3%)	101/257 (39%)		
Roedl [24]	167/223 (75%)	37/167 (22%)	108/167 (64%)	19/167 (11%)	20/223 (9%)	78/223 (35%)		
Auld [25]	165/217 (76%)			22/165 (13%)	4/165 (2%)	62/217 (29%)	8/217 (4%)	147/217 (68%)
Mitra [8]	74/117 (63%)	50/74 (68%)	21/74 (28%)	8/74 (11%)	3/74 (4%)	18/117 (15%)	12/117 (10%)	87/117 (74%)
Pandya [35]	75/75 (100%)					37/75 (49%)		
Cavayas [41]	43/75 (56%)	16/43 (37%)	11/43 (26%)	15/43 (35%)	1 (2%)	17/75 (23%)		58/75 (77%)
Zangrillo [36]	73/73 (100%)	53/70 (76%)	55/72 (76%)		5/73 (7%)	17/73 (23%)	33/73 (45%)	23/73 (32%)
Ziehr [28]	66/66 (100%)	28/66 (42%)	31/66 (47%)	18/66 (27%)	3/66 (5%)	11/66 (17%)	5/66 (8%)	50/66 (76%)
Sinha [27]	39/39 (100%)					17/39 (44%)		
Laverdure [37]	36/36 (100%)		29/36 (81%)	9/39 (25%)	7 (19%)	4/36 (11%)	7/36 (19%)	25/36 (69%)
Bhatraju [29]	18/24 (75%)	7/18 (39%)	5/18 (28%)	5/18 (28%)	0	12/24 (50%)		
Pedersen [38]	17/17 (100%)		5/17 (29%)		0	7/17 (41%)	6/17 (35%)	4/17 (24%)
Liu X [40]	8/8 (100%)					0/8	3/8 (37%)	5/8 (63%)
LUNGSAFE [50]		21.7%	7.9%	7.7%	3.2%	35.3%		

Data are number of patients (% of the subgroup). NMBA, neuromuscular blocking agent; ECMO, extracorporeal membrane oxygenation; ICU intensive care unit

Prone position was used in up to 81% [37] of the patients and neuromuscular blocking agents were administered to up to 88% [17] of the patients. Higher proportions of patients received these two rescue therapies in European studies relative to North American studies.

Treatment with pulmonary vasodilators (usually inhaled nitric oxide) was reported in only 10 papers, and these treatments were provided to between 11 and 35% of the patients [8, 20, 21, 24, 41].

Use of Extracorporeal Membrane Oxygenation as rescue therapy for refractory hypoxemia ranged from 1 to 19% [23, 37] among the studies.

## Discussion

This review highlights the paucity of data regarding one of the greatest challenges in managing patients with COVID-19 – mechanical ventilation. Extrapolation from the number of patients with confirmed disease suggests that between one quarter to half a million COVID-19 patients have already undergone mechanical ventilation worldwide. Since the pandemic outbreak, more than 80,000 papers have been published regarding COVID-19[42]. Several observational studies have described very large populations of critically ill patients with COVID-19 but these provided little to no information on respiratory mechanics or management of mechanical ventilation [6, 9, 43]. Our search of the literature revealed only 26 studies for a total of 14,075 patients that reported the respiratory mechanics, ventilation settings and parameters of gas exchange in critically ill patients with COVID-19 undergoing IMV. We also identified three review papers that discussed mechanical ventilation of COVID-19 patients [44–46]. However, these reviews did not describe precise ventilator settings or provide measurements of respiratory mechanics.

Oxygenation, or more specifically the PaO_2_/FiO_2_ ratio, was described in all but two of the papers. Although PEEP was reported in most studies, only two described how this parameter was selected, with one study titrating on oxygenation [37] and the other using the PEEP/FiO2 table [28]. In contrast, only a handful of studies described mechanics and ventilation parameters – respiratory system compliance, driving pressures, plateau pressures, PaCO_2_, TV and RR were inconsistently reported. Those values that have been reported raise important questions on the validity of our early prior assumptions regarding these disease characteristics.

In the early stage of the pandemic and based on physiological data collected from only 16 subjects [47], it was postulated that there are two distinct clinical types of COVID-19 respiratory disease, differing in static respiratory system compliance, intrapulmonary shunt fraction and recruitability [48]. It was also suggested that the mechanical ventilation of patients classified to these two clinical types of disease should also differ; those with stiff lungs should be ventilated in accordance with the recommendations for ARDS, and those with compliant lungs may be ventilated with higher tidal volumes (7–9 ml/kg ideal body weight) and lower PEEP (< 10 cmH_2_O) than recommended [49]. Subsequent studies conducted in larger patient populations did not confirm this observation [17, 19, 20, 23, 26]. Our review of the available literature, limited as it is to pooled data from multiple studies with different methodologies, also does not support the existence of a clinical dichotomy. Instead, there appears to be a broad-range continuum. In particular, the median values of Crs ranged from 24 to 49 ml/cmH_2_O [27, 34, 39], narrowing only slightly in studies that included more than 100 patients. In addition, when only studies that reported the median (IQR) Crs are considered, only 21% of the 75th percentile values are higher than 50 ml/cmH_2_O. These values are comparable to those reported in the LUNG SAFE study, where Crs varied from 37 (28–53) ml/cmH_2_O in mild ARDS to 28 (22–39) ml/cmH_2_O in severe ARDS [50].

The settings used for mechanical ventilation were relatively consistent across the studies and generally followed evidence-based recommendations for lung protective ventilation [51]. Plateau and driving pressure were largely within the protective limits, with median Pplat values that ranged from 20.5 to 31 cmH_2_O [27, 34] and median driving pressures from 9.5 to 15 cmH_2_O [20, 32, 39]. Median tidal volume almost never exceeded 8 ml/Kg and in most studies it was set around the recommended value of 6 ml/kg PBW [51]. However, since PaCO_2_ and RR were inconsistently reported it remains unclear how these were modified to accommodate the required volume and pressure limits in the subset of patients with reduced lung compliance.

In all but two studies, the median PEEP level was ≥ 10 cmH_2_O. These values are somewhat higher than those reported in the LUNG SAFE study (8.4 cmH_2_O) [50]. COVID-19 patients are often severely hypoxemic at presentation (baseline PaO_2_/FiO_2_ frequently < 150 mmHg) which may explain the application of these levels of PEEP and the higher rates of prone positioning and neuromuscular blocking agents compared to “classical” ARDS patients (respectively ranging from 17 to 81 [20, 37] and 22 to 88% [17, 24] versus 7.9% and 21.7% in the LUNG SAFE study [50]). However, in the absence of detailed information regarding the exact distribution of the PaO_2_/FiO_2_ measurements and their relationship with PEEP all comparisons may be moot.

The principal aim of scoping reviews is to highlight knowledge gaps and, in this regard, the current review provides justification for additional studies of mechanical ventilation of critically ill COVID-19 patients. We found no studies comparing different ventilation strategies (e.g. different approaches to PEEP titration) and few studies (5/26) that reported the mode of ventilation [18, 23, 26, 31, 36]. Only 5/26 studies reported mechanical power which is a critical parameter [17, 18, 23, 41]. Such information could prove extremely important for guiding the mechanical ventilation of patients with COVID-19. Only a handful of small studies, performed in highly selected patient populations, attempted to characterize the mechanical properties of the respiratory system in COVID-19 patients undergoing invasive mechanical ventilation using advanced monitoring techniques (e.g. esophageal pressure monitoring, electrical impedance tomography) [30, 52, 53]. The pathophysiological mechanisms underlying the severe hypoxemia observed in patients with COVID 19 have not been elucidated; in particular, the respective contributions of ventilation-perfusion mismatch, and the dysregulation of hypoxic vasoconstriction and occlusion of the pulmonary vascular bed require further study. Finally, the role of rescue therapies, such as prone positioning and ECMO, in the treatment of refractory hypoxemia, and their actual impact on patient outcomes remain unclear; however the higher rate of prone positioning compared to LUNG SAFE and a better adherence to protective ventilation strategies are substantial findings clearly pointed out by the present review.

Our review has several strengths. To the best of our knowledge, this is the first scoping review on respiratory mechanics and ventilation settings in critically ill patients with COVID-19. The methodology of a scoping review enables comprehensive mapping of current knowledge and identification of knowledge gaps in the existing literature. We used predefined inclusion and exclusion criteria and adhered to the PRISMA ScR checklist to ensure consistency in reviewer agreement, data extraction and synthesis.

We also acknowledge some significant limitations of our work. A scoping review is only as good as the studies it identifies. Most of the studies included in the present review were retrospective, and the few conducted prospectively were observational. The studies are highly heterogeneous with regard to the number of variables analyzed and the quality of the data they present. For example, two of the studies with the largest samples reported only the value of PEEP [21, 22]. None of the studies evaluated how spontaneous breathing may have influenced respiratory mechanics measurements, however, since the majority of the patients were paralyzed with continuous infusion of NMBA (up to 88%) the impact of spontaneous breathing activity should be null or extremely limited. The variability in the type and quality of data presented and the abundance of missing data probably reflect the many difficulties encountered by researchers endeavoring to collect data on these complex patients. COVID ICUs were created in an impromptu manner. Clinicians workload was overwhelming; makeshift equipment with no interface with the hospital electronic medical records was often placed in these units, effectively reducing availability of electronic documentation. The nursing staff working in such units may have had little ICU training, which also limited the quality of documentation. Additionally, some of the studies we identified were based on manual review of medical records, which has inherent limitations [8, 21, 25]. Quantitative data were sometimes reported as medians and other times as means. All of these issues make study comparisons challenging and preclude the pooling of findings, resulting in an inability to draw definitive conclusions.

We also excluded studies reporting respiratory mechanics after the first 24 h of ICU admission. This approach was used to reduce data heterogeneity and the need to account for missing data, but also resulted in a lack of information regarding the evolution of the respiratory disease. As an example, few studies have evaluated the recruitability of COVID-19 patients. These studies could not be included in this review as their design and characteristics did not fulfill the inclusion criteria. However, those studies show that the potential for lung recruitment in COVID-19 is also highly heterogeneous, similar to the range observed in “classical” ARDS patients [52–55].

## Conclusion

The available literature shows that critically ill COVID-19 patients requiring invasive ventilation have very heterogeneous gas exchange and respiratory mechanics during the first 24 h of ICU admission. This finding is reminiscent of the characteristics reported in patients with ARDS from other causes. Low tidal volumes and levels of PEEP equal to or higher than 10 cmH_2_O are commonly used. Prone positioning is more frequently used than in other causes of ARDS. We identified significant gaps in current knowledge, particularly regarding the mechanical properties of the respiratory system, the relative contribution of different pathophysiological mechanisms to the generation of hypoxemia, the optimal settings of mechanical ventilation, the potential for lung recruitment, the response to different PEEP levels and the role of rescue treatments for refractory hypoxemia. Our review highlights the need for a pooled analysis of available data to further fill these knowledge gaps.

## Supplementary Information

**Additional file 1**. Preferred Reporting Items for Systematic reviews and Meta-Analyses extension for Scoping Reviews (PRISMA-ScR) Checklist. Description of data: compiled PRISMA-ScR checklist.

**Additional file 2**. Online search strategy; Table S1; Table S2; Table S3. Description of data: additional file 2 contains the full online search strategy, Table S1 summarizing the characteristics of the included studies, Table S2 assessing the quality of the included studies and table S3 reporting the inclusion and exclusion criteria of the present review.

## Data Availability

All data generated or analysed during this study are included in this published article (and its supplementary information files).

## Abbreviations

ICU: Intensive care unit
COVID-19: Coronavirus disease 2019
IMV: Invasive mechanical ventilation
VILI: Ventilator-induced lung injury
ARDS: Acute respiratory distress syndrome
OSF: Open Science Framework
PRISMA-ScR: Preferred Reporting Items for Systematic reviews and Meta-Analyses extension for Scoping Reviews
ECMO: Extracorporeal membrane oxygenation
PEEP: Positive end-expiratory pressure
TV/Pbw: Tidal volume in relation to predicted body weight
Pplat: Plateau pressure
ΔP: Driving pressure
Crs: Compliance of the respiratory system
RR: Respiratory rate
PaO_2_: Partial pressure of arterial oxygen
FiO_2_: Fraction of inspired oxygen
PaCO_2_: Partial pressure of arterial carbon dioxide
IQR: Interquartile range
SD: Standard deviation
