# Supplementary material for: Mechanical ventilation parameters in critically ill COVID-19 patients: a scoping review
Source: Crit Care. 2021 Mar 20;25:115. doi: 10.1186/s13054-021-03536-2 (PMC7980724; doi:10.1186/s13054-021-03536-2)
Supplement: Supplementary file 2 — Additional file 2. Online search strategy; Table S1; Table S2; Table S3. Description of data: additional file 2 contains the full online search strategy, Table S1 summarizing the characteristics of the included studies, Table S2 assessing the quality of the included studies and table S3 reporting the inclusion and exclusion criteria of the present review. [file 13054_2021_3536_MOESM2_ESM.docx]

**Supplementary material 2**

**Search strategy used for the searches (using EMBASE search engine**): 'respiratory mechanics'/exp OR 'respiratory mechanics' OR 'mechanical ventilation'/exp OR 'mechanical ventilation' OR 'invasive ventilation'/exp OR 'invasive ventilation' OR 'acute respiratory failure'/exp OR 'acute respiratory failure' OR 'adult respiratory distress syndrome'/exp OR 'adult respiratory distress syndrome' OR 'ards'/exp OR 'ards') AND ('coronavirus disease 2019'/exp OR 'coronavirus disease 2019' OR 'severe acute respiratory syndrome coronavirus 2'/exp OR 'severe acute respiratory syndrome coronavirus 2' OR 'covid*' OR 'sars coronavirus'/exp OR 'sars coronavirus' OR 'sarscov*') AND ([embase]/lim OR [medline]/lim OR [pubmed-not-medline]/lim

**Table S1**:summary of the charateristics of the included studies

| **Author** | **ID** | **Country** | **Design** | **Participants (No.)** | **IMV (No.)** | **Outcomes** | **Results** |
| --- | --- | --- | --- | --- | --- | --- | --- |
| **Schmidt et al.** | NA | France, Belgium, and  Switzerland | Multicenter prospective observational cohort study | All consecutive adults with laboratory-confirmed COVID-19 admitted to intensive care unit (4244) | 3376 | Demographic and clinical data, risk factors associated with 90-day mortality. | On day 90, 1298/4244 (31%) patients had died. Among patients who received invasive or noninvasive ventilation on the day of ICU admission, day-90 mortality increased with the severity of ARDS at ICU admission and decreased from 42 to 25% over the  study period. Early independent predictors of 90-day mortality were older age, immunosuppression, severe obesity,  diabetes, higher renal and cardiovascular SOFA score components, lower PaO2/  FiO2 ratio and a shorter time between  first symptoms and ICU admission. |
| **Gupta et al.** | NA | USA | Multicenter retrospective observational cohort study | All consecutive adults with laboratory-confirmed COVID-19 admitted to intensive care unit (2215) | 1859 | Primary: 28 day in-hospital mortality, Secondary: respiratory failure, ARDS, congestive heart failure, myocarditis, pericarditis, arrhythmia, shock, acute cardiac injury, acute kidney injury, acute liver injury, coagulopathy, secondary  infection, and thromboembolic event | At 28 days after ICU admission, 784 patients (35.4%) had died, 824 (37.2%) were discharged, and 607 (27.4%) remained hospitalized. Factors independently associated with death included older age, male sex, higher body mass index (>40 vs <25), coronary artery disease, active cancer, and the presence of hypoxemia, liver dysfunction and kidney dysfunction at ICU admission |
| **Grasselli et al.** | NA | Italy | Multicenter retrospective observational cohort study | All consecutive adults with laboratory-confirmed COVID-19 admitted to intensive care unit (3988) | 2929 | Demographic and clinical data, in-hospital mortality. | Median age was 63 years; 3188 (79.9%; 95%CI, 78.7%-81.1%) were men, and  1998 of 3300 (60.5%; 95%CI, 58.9%-62.2%) had at least 1 comorbidity. At ICU admission,  2929 patients (87.3%; 95%CI, 86.1%-88.4%) required invasive mechanical ventilation. In the subgroup of the first 1715 patients, as of May 30, 2020, 865 (50.4%) had been discharged from the ICU, 836 (48.7%) had died in the ICU, and 14 (0.8%) were still in  the ICU |
| **Ferrando et al.** | NCT04368975 | Spain | Multicenter prospective observational cohort study | All consecutive mechanically ventilated patients with COVID-19 ARDS (742) | 742 | Primary: describe the physiologic characteristics over time, the ventilatory management, and outcomes in ARDS COVID-19 patients. Secondary: to compare respiratory parameters and outcomes of ARDS COVID-19 patients with ARDS of other causes. | Values of respiratory system compliance, plateau pressure, and driving pressure were similar to values from non-COVID-19 ARDS observed in other studies. The 28-day mortality (32%) was similar to other observational studies in non-COVID-19 ARDS patients. |
| **Botta et al.** | NCT04346342 | Netherlands | Multicenter retrospective observational cohort study | All consecutive mechanically ventilated patients with laboratory-confirmed COVID-19 (553) | 553 | Primary: a combination of ventilator variables and parameters over the first 4 calendar days of ventilation: tidal volume, positive end-expiratory pressure, respiratory system compliance, and driving pressure. Secondary: the use of adjunctive treatments for refractory hypoxaemia and ICU complications. Patient-centred outcomes were ventilator-free days at day 28, duration of ventilation, duration of  ICU and hospital stay, and mortality | Of the adjunctive treatments for refractory hypoxemia, prone positioning was most often used in the first 4 days of ventilation (53%). The median number of ventilator-free days at day 28 was 0 (IQR 0–15); 186 (35%) of 530 patients had died by day 28. Predictors of 28-day mortality were gender, age, tidal volume, respiratory system  compliance, arterial pH, and heart rate on the first day of invasive ventilation. |
| **Grasselli et al. (LRM)** | NA | Italy | Multicenter prospective observational cohort study | All consecutive mechanically ventilated patients with laboratory-confirmed COVID-19, admitted to intensive care unit (338) | 301 | To characterize COVID-19-induced ARDS and compare it to ‘classical’ ARDS. 28 day in-hospital mortality | Patients with static compliance equal to or  less than the median and D-dimer concentrations greater than the median had markedly increased 28-day mortality  compared with other patient subgroups. 28-day mortality was 36% |
| **Schenck et al.** | NA | USA | Single center prospective observational cohort study | All consecutive mechanically ventilated patients with laboratory-confirmed COVID-19 (267) | 267 | Physiologic and clinical information during the first week of mechanical ventilation and in-hospital mortality | During the observed time period, 77 patients were successfully extubated and 49 died. On day 3 the minute ventilation was higher in those that died compared to those who don’t die. |
| **Cummings et al.** | NA | USA | Multicenter prospective observational cohort study | Adults with laboratory-confirmed COVID-19 admitted to hospital and critically ill (257) | 203 | Primary: in-hospital mortality. Secondary: frequency and duration of invasive mechanical ventilation, frequency of vasopressor use and renal replacement therapy, and time to in-hospital clinical deterioration following admission | At final follow up 101 (39%) patients had died and 94 (37%) remained hospitalised. 203 (79%) patients received invasive mechanical ventilation for a median of 18 days (IQR 9–28), 170 (66%) of 257 patients received vasopressors and 79 (31%) received renal replacement therapy. The  median time to in-hospital deterioration was 3 days (IQR 1–6). |
| **Roedl et al.** | NA | Germany | Multicenter retrospective observational cohort study | All consecutive adults with laboratory-confirmed COVID-19 admitted to intensive care unit (223) | 167 | Demographic and clinical data, in-hospital mortality. | A total of 223 critically ill patients with COVID-19 were included. The majority, 73%, were men; the median age was 69 years, with 68% patients having at least one chronic medical condition. Overall, 167 (75%) patients needed mechanical ventilation. The ICU mortality was 35% and 44% among mechanically ventilated patients. |
| **Auld et al.** | NA | USA | Multicenter retrospective observational cohort study | All consecutive adults with laboratory-confirmed COVID-19 admitted to intensive care unit (217) | 165 | In-hospital mortality | Among the 217 patients in the cohort, 147 (67.7%) have been transferred alive from the ICU, 62 died (28.6%) in the ICU, and 8 (3.7%) remain in the ICU, all of whom are still  mechanically ventilated. |
| **Mitra et al.** | NA | Canada | Multicenter retrospective observational cohort study | All consecutive adults with laboratory-confirmed COVID-19 admitted to intensive care unit (117) | 74 | Clinical  characteristics and in-hospital mortality | The mortality for patients with an ICU outcome was 18 of 105 (17.1%). Of the 74 patients who underwent mechanical ventilation, 12 (16.2%) remained in ICU, 13 (17.6%) were discharged from ICU but remained in hospital, 34 (45.9%) were discharged home, and 15 (20.3%) died. |
| **Pandya et al.** | NA | USA | Single center retrospective observational case series. | All consecutive mechanically ventilated patients with laboratory-confirmed COVID-19 (75) | 75 |  | In hospital mortality was of 49%. Nonsurvivors had higher initial FIO2 requirement, lower PaO2/FIO2 ratio, lower  static compliance, and higher ventilatory ratios. |
| **Zangrillo et al.** | NA | Italy | Single center retrospective observational case series | Mechanically ventilated patients with laboratory confirmed COVID-19 admitted to the intensive care unit (73) | 73 | Clinical  characteristics and in-hospital mortality. | After a median follow up of 19.0 days (IQR, 15.0–27.0 days), 17 patients (23.3%) had died, 23 (31.5%) had been discharged from the ICU, and 33 (45.2%) were receiving invasive mechanical ventilation in the ICU. Older age (odds ratio [OR], 1.12; 95% CI, 1.04–1.22; P = 0.004) and hypertension (OR, 6.15; 95% CI, 1.75–29.11; P = 0.009) were associated with mortality |
| **Ziehr et al.** | NA | USA | Multicenter retrospective observational case series | Mechanically ventilated patients with laboratory-confirmed COVID-19 (66) | 66 | Respiratory pathophysiology of patients with COVID-19 respiratory failure and in-hospital mortality. | Forty-one patients (62.1%) were successfully extubated, among whom the median duration of mechanical ventilation was 16.0 days (IQR, 10.0-21.0). Fifty patients (75.8%) were discharged from the ICU. Eleven patients (16.7%) died. |
| **Sinha et al.** | NA | UK | Multicenter prospective observational case series | All consecutive patients with laboratory confirmed COVID-19 and ARDS | 39 | Describe the prevalence of ARDS phenotypes in COVID-19 associated ARDS. Compare the clinical and biological characteristics of patients with COVID-19 and ARDS to a previously characterised population of patients  with ARDS due to other causes | Depending on the probability cutoff used to assign class, the prevalence of the hyperinflammatory phenotype was between four (10%) and eight (21%) of 39, which is  lower than the proportion of patients with the hyperinflammatory phenotype in HARP-2 (35%). Using the Youden index cutoff (0·274) to classify phenotype, five (63%) of eight patients with the hyperinflammatory  phenotype and 12 (39%) of 31 with the hypoinflammatory phenotype died. 17 (44%) of 39 patients had died by day 28 of the  study. |
| **Laverdure et al.** | NA | France | Single center retrospective observational case series | Mechanically ventilated patients with laboratory-confirmed COVID-19 (36) | 36 | Describe characteristics  and outcomes in mechanically ventilated COVID-19 patients | The median baseline Cst-rs was 36 mL/cmH2O. On day 28, 32 patients (88.9%) survived and 25 (69.4%) were discharged from the intensive care unit and 4 (11%) died |
| **Bos et al.** | NA | Netherlands | Single center retrospective observational case series | Adults with suspected COVID-19 admitted to intensive care unit (38) | 38 | Respiratory physiology and CT scan of patients with suspected COVID-19 | There was no relation between Crs and poorly or non-aerated lung tissue. Most patients had a non-focal lung morphology (N=30, 79%). Patients with a non-focal lung morphology had more parenchymal involvement (P=0.0065), but not a lower Crs (P=0.72) than patients with focal lung morphology |
| **Haudebourg et al.** | NA | France | Single center prospective observational case series | All consecutive adults with laboratory-confirmed COVID-19 and ARDS (30) | 30 | The respiratory mechanics and lung recruitability of COVID-19 associated ARDS patients, to compare it to that of non-COVID-19 ARDS. | Driving pressure as well as respiratory system compliance and resistance did not significantly differ between COVID-19 and non-COVID-19 patients. the R/I ratio was significantly higher in COVID-19 than in non-COVID-19 patients |
| **Beloncle et al.** | NCT04350710 | France | Single center prospective observational case series | All consecutive adults with laboratory-confirmed COVID-19 and ARDS within 24 hours from intubation (25) | 25 | The respiratory mechanics and lung recruitability | At day 1, 16 (64%) were considered as highly recruitable and 9 (36%) were  considered as poorly recruitable. |
| **Bhatraju et al.** | NA | USA | Multicenter retrospective observational case series | Adults with laboratory-confirmed COVID-19 admitted to intensive care unit (24) | 18 | The demographic characteristics  and in-hospital mortality | 75% of the patients needed mechanical ventilation. 50% of the patients died. Of the 12 surviving patients, 5 were discharged home, 4 were discharged from the ICU but remained in the hospital, and 3 continued to receive mechanical ventilation in the ICU |
| **Cavayas YA et al.** | NA | Canada | Single center retrospective observational case series | All consecutive adults with laboratory-confirmed COVID-19 admitted to intensive care unit (75) | 43 | Describe the demographics, presentation, treatments, and outcomes of a cohort of critically ill adult patients with COVID-19 | Patients receiving IMV were characterized by a  moderately decreased median [IQR] PaO_2_/FIO_2_ (day 1 PaO_2_/FIO_2_ = 177 [138-276]) and compliance (day 1 = 48 [38-58] mL/cmH_2_O) and very elevated estimated dead space fraction  (day 1 = 0.60 [0.53-0.67]). Overall hospital mortality was 25%, and 21% in the IMV patients. |
| **Diehl et al.** | NA | France | Single center prospective observational case series | All consecutive adults with COVID-19 ARDS without history of chronic respiratory disease (22) | 22 | Respiratory mechanics and gas exchanges | We observed moderately decreased respiratory system compliance and end-expiratory lung volume. Gas exchanges were  characterized by hypercapnia 55 [44–62] mmHg, high physiological dead-space (VD/VT): 75 [69–85.5] % and ventilatory ratio (VR): 2.9 [2.2–3.4]. |
| **Pedersen et al.** | NA | Denmark | Single center retrospective observational case series | Adults with COVID-19 admitted to intensive care unit (17) | 17 | Clinical characteristics and in-hospital mortality | By 16 April, six patients were still admitted to the ICU, four patients had been discharged from the ICU and seven had died. |
| **Roesthuis et al.** | NA | Netherlands | Single center prospective observational case series | Adults with COVID-19 admitted to intensive care unit (14) | 14 | Respiratory mechanics of mechanically ventilated COVID-19 patients | COVID-19 patients had high dead space ventilation and gas exchange impairment (Bohr 52 ± 3%; Enghoff modification 67 ± 2%). Reducing PEEP resulted in an increase in lung compliance and decrease in dead space ventilation |
| **Carsetti et al.** | NA | Italy | Single center retrospective case series | Adults with COVID-19 admitted to intensive care unit and mechanically ventilated (10) | 10 | Feasibility of prolonged prone position | Prolonged prone position up to 36 h is feasible, safe, and may offer potential  clinical and organizational advantages |
| **Liu X et al.** | NA | China | Single center retrospective observational case series | Adults with COVID-19 admitted to intensive care unit and mechanically ventilated (8) | 8 | Ventilatory ratio in hypercapnic mechanically ventilated patients with COVID-19 | Ventilatory ratio was measured, the mean value was 2.1 ± 0.3 in the initial four patients, suggesting high VD/VT. An increased VT (7.7 ± 0.8 ml/kg PBW) was applied VR in the four patients was significantly decreased (1.760.2 vs. 2.160.3; P = 0.018) and PaO2/FIO2 was slightly improved (241 ± 38 mm Hg vs. 207 ± 61; P = 0.402). |

**Table S2**: Quality assesment of the incuded studies using the Newcastle Ottawa score for cohort studies

| Study | **Case Control / Cohort** | **Selection** | | | | **Comparability** | **Exposure / Outcome** | | | **Score** |
| --- | --- | --- | --- | --- | --- | --- | --- | --- | --- | --- |
|  |  | **1** | **2** | **3** | **4** | **1** | **1** | **2** | **3** |  |
|  |  |  |  |  |  |  |  |  |  |  |
| *Schmidt M et al. Intensive Care Med. 2020 Oct 29;1-14* | Cohort | * |  | * | * |  | * | * | * | 6 |
| *Grasselli G et al. JAMA Intern Med. 2020;e203539.* | Cohort | * |  | * | * |  | * | * | * | 6 |
| *Gupta S et al. JAMA Intern Med. 2020;e203596.* | Cohort | * |  | * | * |  | * | * | * | 6 |
| *Ferrando C et al. Intensive Care Med. 2020;1-12.* | Cohort | * |  | * | * |  | * | * | * | 6 |
| *Botta M et al. Lancet Respir Med 2020 Oct23;S2213-2600(20)30459-8* | Cohort | * |  | * | * |  | * | * | * | 6 |
| *Grasselli G et al. Lancet Respir Med. 2020;S2213-2600(20)30370-2.* | Cohort | * |  | * | * |  | * | * | * | 6 |
| *Schenck EJ et al. Ann Am Thorac Soc. 2020;10.1513/AnnalsATS.202005-427RL.* | Cohort | * |  | * | * |  | * |  | * | 5 |
| *Cummings MJ et al. Lancet. 2020;395(10239):1763-1770.* | Cohort | * |  | * | * |  | * | * | * | 6 |
| *Roedl K et al. Aust Crit Care. 2020 Oct 27;S1036-7314(20)30334-9* | Cohort | * |  | * | * |  | * | * | * | 6 |
| *Auld SC et al. Crit Care Med. 2020;48(9):e799-e804.* | Cohort | * |  | * | * |  | * |  | * | 5 |
| *Mitra AR et al. CMAJ. 2020;192(26):E694-E701.* | Case Series | * |  | * | * |  | * |  | * | 5 |
| *Pandya A et al.* *Chest. 2020 Aug 31;S0012-3692(20)34314-2* | Case Series | * |  | * | * |  | * | * |  | 5 |
| *Cavayas AY et al.* *Can J Anaesth. 2020 Sep 15;1-10* | Case Series | * |  | * | * |  | * | * | * | 6 |
| *Zangrillo A et al. Crit Care Resusc. 2020;* | Case Series | * |  | * | * |  | * |  | * | 5 |
| *Ziehr DR et al. Am J Respir Crit Care Med. 2020;201(12):1560-1564* | Case Series | * |  | * | * |  | * | * | * | 6 |
| *Sinha P et al. Lancet Respir Med. 2020;S2213-2600(20)30366-0.* | Case Series | * |  | * | * |  | * | * | * | 6 |
| *Laverdure F et al. Crit Care. 2020 Jul 9;24(1):412* | Case Series |  |  | * | * |  | * | * | * | 5 |
| *Bos LD et al. Ann Am Thorac Soc. 2020;10.1513/AnnalsATS.202004-376RL* | Case Series | * |  |  |  |  |  |  |  | 1 |
| *Haudebourg AF et al. Am J Respir Crit Care Med.2020;202(2):287-290* | Case Series | * | * | * | * | ** | * | * | * | 9 |
| *Beloncle FM et al. Ann Intensive Care. 2020;10(1):55.* | Case Series | * |  | * | * |  | * | * |  | 5 |
| *Bhatraju PK et al. N Engl J Med. 2020;382(21):2012-2022.* | Case Series | * |  | * | * |  | * |  | * | 5 |
| *Diehl JL et al. Ann Intensive Care. 2020;10(1):95.* | Case Series |  |  | * | * |  | * |  |  | 3 |
| *Pedersen HP et al. Dan Med J. 2020;67(5):A04200232.* | Case Series | * |  | * | * |  | * |  | * | 5 |
| *Roesthuis L et al. Crit Care. 2020;24(1):230.* | Case Series | * |  | * | * |  | * | * | * | 6 |
| *Carsetti A et al. Crit Care. 2020;24(1):225.* | Case Series |  |  | * | * |  | * | * |  | 4 |
| *Liu X et al. Am J Respir Crit Care Med. 2020;201(10):1297-1299.* | Case Series |  |  | * | * |  | * | * |  | 4 |

**Table S3:** summary of the inclusion and exclusion criteria of the present review

| Inclusion Criteria | Exclusion Criteria |
| --- | --- |
| Age ≥ 18 | Studies in language other than english |
| Diagnosis of COVID-19 | Non-Invasive Ventilation |
| Invasive Mechanical Ventilation in ICU | Case reports |
| Studies providing at least one parameter of respiratory mechanics at ICU admission or within the first day of ICU stay | Review of the literature |
| Studies providing at least one parameter of ventilator setting at ICU admission or within the first day of ICU stay | Patients treated with ECMO |
